# Profiles of Adolescent Social Media Use Intensity and Subjective Well-Being: The Sequential Mediating Roles of Upward Social Comparison and Body Appreciation

**DOI:** 10.3390/bs16091567

**Published:** 2026-09-04

**Authors:** Yaxin Yang, Yunrui Wang, Na Zhang, Mei Xu, Xiaozhu Ling, Jianhua Wang, Xuemei Gao

**Affiliations:** 1 Psychological Research and Counseling Center, Southwest Jiaotong University, Chengdu 611756, China; yangyaxin@my.swjtu.edu.cn (Y.Y.); wangyunrui@my.swjtu.edu.cn (Y.W.); 18301170130@fudan.edu.cn (N.Z.); xumei00@my.swjtu.edu.cn (M.X.); lingxiaozhu@my.swjtu.edu.cn (X.L.); 2 Panzhou No.2 Middle School, Panzhou 553537, China; zn2024201650@my.switu.edu.cn

**Keywords:** adolescents, social media use intensity, subjective well-being, upward social comparison, body appreciation

## Abstract

The relationship between social media use and adolescent well-being remains complex, and the psychological processes involved in this association require further investigation. Using data from 2085 Chinese adolescents aged 10–19 years, this study identified three social media use intensity profiles through latent profile analysis and examined whether upward social comparison and body appreciation formed a sequential mediating pathway linking social media use intensity and subjective well-being. Three profiles were identified: low social media use intensity (25.2%), moderate social media use intensity (59.0%), and high social media use intensity (15.8%) profiles. In the overall sample, the sequential indirect effect from social media use intensity through upward social comparison and body appreciation to subjective well-being was significant. Furthermore, subgroup-specific analyses showed that the total effect, direct effect, and total indirect effect were not significant in any of the three profiles. A specific sequential indirect pathway through upward social comparison and body appreciation was significant only in the high social media use intensity profile. Moreover, the negative association between upward social comparison and body appreciation was significantly stronger in the high social media use intensity profile than in both the low and moderate social media use intensity profiles.

## 1. Introduction

Social media has become an important context for adolescents’ daily social interactions and self-expression (Orben et al., 2022; Twenge et al., 2017). Adolescents widely use platforms such as Instagram, TikTok, and Weibo for interpersonal communication, self-presentation, entertainment, and information seeking. Unlike traditional media, social media not only facilitates information dissemination but also provides a dynamic environment in which social interactions, identity exploration, and social feedback continuously occur. Within this environment, adolescents are exposed to abundant information regarding others’ lifestyles, social performance, and social evaluations.

Adolescence represents a critical developmental period for the formation of self-concept and identity. According to Erikson’s theory of psychosocial development, adolescents are in the stage of “identity versus role confusion,” during which they gradually develop a stable sense of self through social interactions and feedback from significant others (Erikson, 1968). Given the important role of social evaluation in identity development, adolescents typically exhibit heightened sensitivity to social evaluation (Somerville, 2013). Meanwhile, adolescence remains a period of ongoing development in social cognition and self-regulation. Therefore, as a highly socialized information environment, social media may be associated with adolescents’ subjective well-being by shaping how they process socially evaluative information.

This process may be particularly relevant among Chinese adolescents. Within a relationship-oriented cultural context, individuals tend to understand and evaluate themselves through feedback derived from social relationships (Markus & Kitayama, 1991). Accordingly, adolescents may place greater emphasis on peer evaluations and social comparison information. From the perspective of social comparison theory, individuals have a fundamental tendency to evaluate their abilities, worth, and social standing through comparisons with others (Festinger, 1954). By increasing the accessibility of social information, social media enables adolescents to encounter others’ lifestyles and social performances more frequently. Meanwhile, some forms of self-presentation on social media are selective and idealized in nature (Fardouly & Vartanian, 2015; Vogel et al., 2014), making individuals more likely to encounter curated positive information and idealized images.

The Tripartite Influence Model proposed by Thompson et al. (1999) further explains the processes through which sociocultural factors contribute to the development of body image. In recent years, researchers have extended this model to the social media context and found that appearance-related information on social media may influence individuals’ body image through appearance-related social comparisons and the internalization of ideal appearance standards (Jarman et al., 2021; Jung et al., 2022). For adolescents undergoing rapid physical development, appearance gradually becomes an important component of self-evaluation and social interactions. The extensive appearance-related content on social media, including selfies, body displays, and edited visual materials, may increase adolescents’ likelihood of engaging in appearance-related social comparisons. When individuals repeatedly attend to comparison targets perceived as more attractive, they may further reinforce ideal body standards and develop more negative evaluations of their own bodies (Fardouly & Vartanian, 2015). Therefore, upward social comparison may represent an important psychological process underlying adolescents’ body image.

Compared with negative indicators such as body dissatisfaction, body appreciation emphasizes individuals’ positive acceptance and respect toward their bodies, as well as their recognition of body functionality and uniqueness, representing an important aspect of positive body image. Previous research has shown that higher levels of body appreciation are generally associated with more positive emotional experiences, greater life satisfaction, and better psychological health, whereas lower levels of body appreciation may be accompanied by greater body dissatisfaction and negative emotional experiences (Linardon et al., 2022). Therefore, when adolescents experience more negative body-related evaluation processes, their levels of body appreciation may be affected, which may further relate to subjective well-being.

Based on the aforementioned theoretical perspectives, the present study proposes that social media use intensity, upward social comparison, body appreciation, and subjective well-being may constitute a sequential cognitive–evaluative pathway. Specifically, higher levels of social media use intensity may indicate deeper engagement with the social media environment and stronger psychological connections with social information presented on these platforms, which may be associated with greater tendencies toward upward social comparison. When comparison processes involve appearance-related domains, they may undermine individuals’ positive evaluations of their own bodies. Subsequently, lower levels of body appreciation may be associated with lower subjective well-being. Accordingly, upward social comparison and body appreciation may form a sequential mediation pathway linking social media use intensity and subjective well-being.

Although previous studies have examined the relationship between social media use and adolescents’ psychological health, several limitations remain. First, existing research has primarily focused on behavioral indicators of social media use, such as duration and frequency, while paying relatively less attention to the psychological connection between individuals and social media (Orben et al., 2019). Therefore, the present study adopts the concept of “social media use intensity” proposed by Ellison et al. (2007) to examine the psychological importance of social media in adolescents’ daily lives.

Second, previous studies have mainly adopted a variable-centered approach to examine the average associations between social media use and psychological outcomes. However, this approach may be insufficient for identifying potential differences in social media use patterns among individuals (Odgers & Jensen, 2020; Teague et al., 2026). In fact, adolescents may comprise distinct subgroups characterized by different patterns of social media use intensity, and these subgroups may demonstrate different patterns of psychological outcomes. Compared with variable-centered approaches that primarily focus on average associations among variables, person-centered approaches can identify subgroups of adolescents who share similar characteristics in social media use. Rather than treating social media use intensity as a single continuous variable or arbitrarily dividing individuals based on total scale scores, latent profile analysis can identify underlying subgroups based on response patterns across multiple indicators. This approach may provide a more accurate understanding of potential heterogeneity in adolescents’ social media use intensity characteristics and further examine differences in psychological outcomes across subgroups.

Furthermore, although previous studies have identified associations among social media use, social comparison, body image, and well-being (Gerson et al., 2016; Teague et al., 2026), the integrated relationship among these variables remains insufficiently understood, particularly among adolescents with diverse developmental characteristics and cultural backgrounds.

Therefore, the present study adopts a person-centered approach and investigates a sample of 2085 Chinese adolescent students. Latent profile analysis is conducted to identify distinct subgroups characterized by different patterns of social media use intensity. Furthermore, this study examines the sequential mediating roles of upward social comparison and body appreciation in the association between social media use intensity and subjective well-being.

The hypotheses of the present study are as follows:

**H1.**  *Adolescents’ social media use intensity can be classified into distinct latent profiles.*

**H2.**  *Adolescents belonging to different social media use intensity profiles exhibit significant differences in upward social comparison, body appreciation, and subjective well-being.*

**H3.**  *Upward social comparison and body appreciation may form a sequential mediation pathway linking social media use intensity and subjective well-being, and the strength of this pathway may differ across social media use intensity profiles.*

## 2. Method

### 2.1. Participants and Procedure

A cluster random sampling method was used in the present study. Three primary and secondary schools in Panzhou City, Guizhou Province, China, were selected as the sampling sites, and students in Grades 5 to 11 were invited to participate in the questionnaire survey. The survey included measures of social media use intensity, upward social comparison, body appreciation, subjective well-being, and demographic information. After excluding questionnaires with excessive missing responses or apparent careless responding, 2085 valid questionnaires were retained, yielding an effective response rate of 90.65%. Among the participants, 1105 were boys (53.0%) and 980 were girls (47.0%). The participants had a mean age of 14.09 years (SD = 1.80), with ages ranging from 10 to 19 years.

The questionnaires were administered collectively at the class level. Before the formal assessment, the researchers provided standardized instructions regarding the study objectives, assessment procedures, and relevant precautions. All participants read an informed consent statement and independently decided whether to participate in the study. Participants completed the questionnaires independently, and the completed questionnaires were collected immediately after completion. All collected data were used exclusively for academic research purposes. This study adhered to the principles of informed consent and participant protection. For underage participants, their parents or legal guardians were also informed about the study and provided consent before their participation. All research procedures were approved by the ethics committee of the researchers’ institution (Ethics Approval No. XL-20260127-0001).

### 2.2. Measures

#### 2.2.1. Social Media Use Intensity

Social media use intensity was assessed using the Social Media Intensity Scale translated by Niu et al. (2016). The scale was adapted from the Social Network Site Intensity Scale developed by Ellison et al. (2007). It consists of six items designed to assess adolescents’ emotional connection with social media and the extent to which social media is integrated into their everyday lives. Participants responded to each item on a 5-point Likert scale, ranging from 1 (strongly disagree) to 5 (strongly agree). All item scores were standardized and summed to generate the social media intensity score, with higher scores indicating greater intensity of social media use. In the present study, the scale demonstrated good internal consistency, with Cronbach’s α = 0.834.

#### 2.2.2. Upward Social Comparison

Upward social comparison was assessed using the upward social comparison subscale of the Iowa–Netherlands Comparison Orientation Measure (Gibbons & Buunk, 1999). This scale was translated by Bai et al. (2013) in their study of social comparison among Chinese middle school students, and this translated version has been widely used in subsequent research. The subscale consists of six items rated on a 5-point Likert scale. The total score reflects individuals’ tendency toward upward social comparison, with higher scores indicating a greater tendency to engage in upward social comparison. In the present study, the scale demonstrated good internal consistency, with Cronbach’s α = 0.838.

#### 2.2.3. Body Appreciation

The Body Appreciation Scale-2 (BAS-2) was originally developed by Tylka and Wood-Barcalow (2015) and has been widely used to assess positive body image. The scale was translated and revised by Ma et al. (2022) for Chinese populations. The original BAS-2 consists of 10 items, and Tylka et al. (2022) developed a 2-item short form (BAS-2SF), which demonstrated a high correlation with the original BAS-2 (rs = 0.93–0.97) and retained its psychometric properties. In the present study, the 2-item short form of the BAS-2 was used. Items were rated on a 5-point Likert scale (1 = never, 5 = always), and the mean score of the items was calculated, with higher scores indicating higher levels of body appreciation. In the present study, Cronbach’s α = 0.795.

#### 2.2.4. Subjective Well-Being

Subjective well-being was assessed using the Index of Well-Being developed by Campbell et al. (1976), which was subsequently translated and revised by X. D. Wang et al. (1999). It has been widely used to assess subjective well-being among Chinese adolescents and college students. The scale consists of nine items comprising two components: the Affect Balance Scale and the Life Satisfaction Questionnaire. The total score was calculated by summing the mean score of the Affect Balance Scale items and the weighted score of the Life Satisfaction Questionnaire item, with a weighting coefficient of 1.1 applied to the latter. The total score ranges from 2.1 to 14.7, with higher scores indicating greater subjective well-being. In the present study, the scale demonstrated good internal consistency, with Cronbach’s α = 0.878.

### 2.3. Statistical Analysis

Descriptive statistics, correlation analyses, and tests for common method bias were conducted using SPSS 26.0. Latent profile analysis (LPA) and serial mediation analyses were conducted using Mplus 8.3. After identifying latent classes of adolescent social media use intensity, the profile classification results were used as grouping variables to further examine whether the effects showed cross-group consistency across the three groups. The data analyses consisted of three parts. First, LPA was conducted to identify profiles of adolescent social media use intensity. The number of latent profiles was gradually increased from a two-profile model to identify the model with the best fit. Model fit was evaluated using the following indices: (1) information criteria, including the Akaike information criterion (AIC), Bayesian information criterion (BIC), and sample-size-adjusted Bayesian information criterion (aBIC), with lower values indicating better model fit; (2) entropy, which evaluates classification accuracy, with higher values indicating more accurate classification; (3) likelihood ratio tests, including the Lo–Mendell–Rubin (LMR) test and bootstrap likelihood ratio test (BLRT), which compare two nested models, where a significant *p* value indicates that the k-profile model provides a better fit than the k−1-profile model (Lubke & Muthén, 2005). The optimal profile solution was then treated as the dependent variable, and the R3STEP command of the robust three-step approach was used to examine the effects of demographic variables on adolescents’ latent social media use classes through logistic regression analysis. Subsequently, the latent profile classification was used as the independent variable, and the BCH method was applied to examine whether adolescents from different social media use intensity profiles differed in upward social comparison, body appreciation, and subjective well-being. Second, a serial mediation model was constructed, with social media use intensity as the independent variable, subjective well-being as the dependent variable, and upward social comparison and body appreciation as mediating variables. Age, BMI, and gender were included as covariates to examine the serial mediating effects of upward social comparison and body appreciation in the relationship between social media use intensity and subjective well-being. The mediation effects were tested using the bootstrap method with 5000 resamples, and an indirect effect was considered significant when the 95% confidence interval did not include zero (DiCiccio & Efron, 1996). Third, multi-group analysis was conducted to further examine whether the sequential mediation pathways were equivalent across different social media use intensity profiles. Specifically, an unconstrained baseline model was first constructed, in which all path coefficients were freely estimated across the three groups. Next, a constrained model was constructed by setting key path coefficients equal across groups. The chi-square difference test (Δχ^2^) was used to determine whether the constraints resulted in a significant deterioration in model fit. A nonsignificant Δχ^2^ indicates that the sequential mediation pathways do not differ significantly across groups and that the sequential mediation model demonstrates cross-group consistency.

### 2.4. Common Method Bias Assessment

Harman’s single-factor test was conducted using an unrotated exploratory factor analysis of all items from the scales used in this study. The results showed that five factors had eigenvalues greater than 1, and the first factor accounted for 24.10% of the variance (<40%). Therefore, substantial common method bias was unlikely to be a major concern in the present study.

## 3. Results

### 3.1. Descriptive Statistics and Correlation Analyses Among Variables

**Figure 1:**
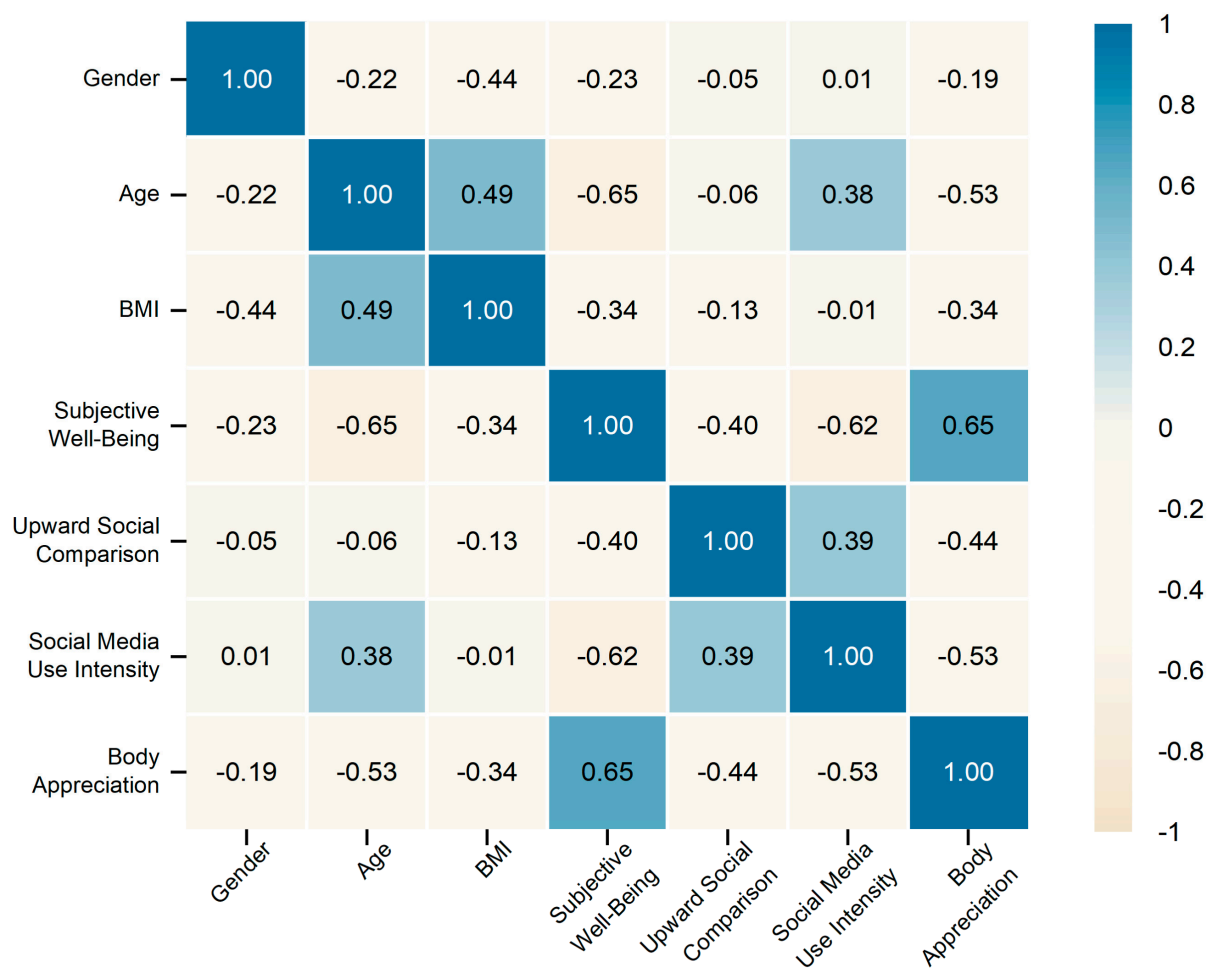
Correlation Matrix of Demographic and Study Variables.

Descriptive statistics and correlation analyses among the study variables are presented in Figure 1. Social media use intensity was significantly positively correlated with upward social comparison and significantly negatively correlated with body appreciation and subjective well-being. Upward social comparison was significantly negatively correlated with both body appreciation and subjective well-being, whereas body appreciation was significantly positively correlated with subjective well-being.

### 3.2. Latent Profile Analysis of Adolescent Social Media Use Intensity

To identify subgroups of adolescent social media use intensity, item-level scores from the Social Media Use Intensity Scale were entered as indicator variables in the latent profile models. Models with two to five latent profiles were compared, and the model fit indices are presented in Table 1. The LPA results indicated that, as the number of profiles increased, the AIC, BIC, and aBIC values decreased from the two-profile to the three-profile model, and the three-profile model exhibited the highest entropy value. Furthermore, the likelihood ratio tests indicated that both the LMR-LRT and BLRT were significant for the three-profile model. Although the AIC, BIC, and aBIC values continued to decrease in the four- and five-profile models, these models showed lower entropy values than the three-profile model and included smaller classes (approximately 9% of the sample). Considering the model fit indices, entropy, class size, and model parsimony, the three-profile model was selected as the final solution. The three-profile model consisted of three classes with proportions of 25.2%, 59.0%, and 15.8%, respectively, and the proportion of each class exceeded the recommended threshold of 10%, supporting the interpretability of the three-profile solution. Taken together, these indices supported the three-profile model as the optimal solution for characterizing adolescent social media use intensity in the present study.

**Figure 2:**
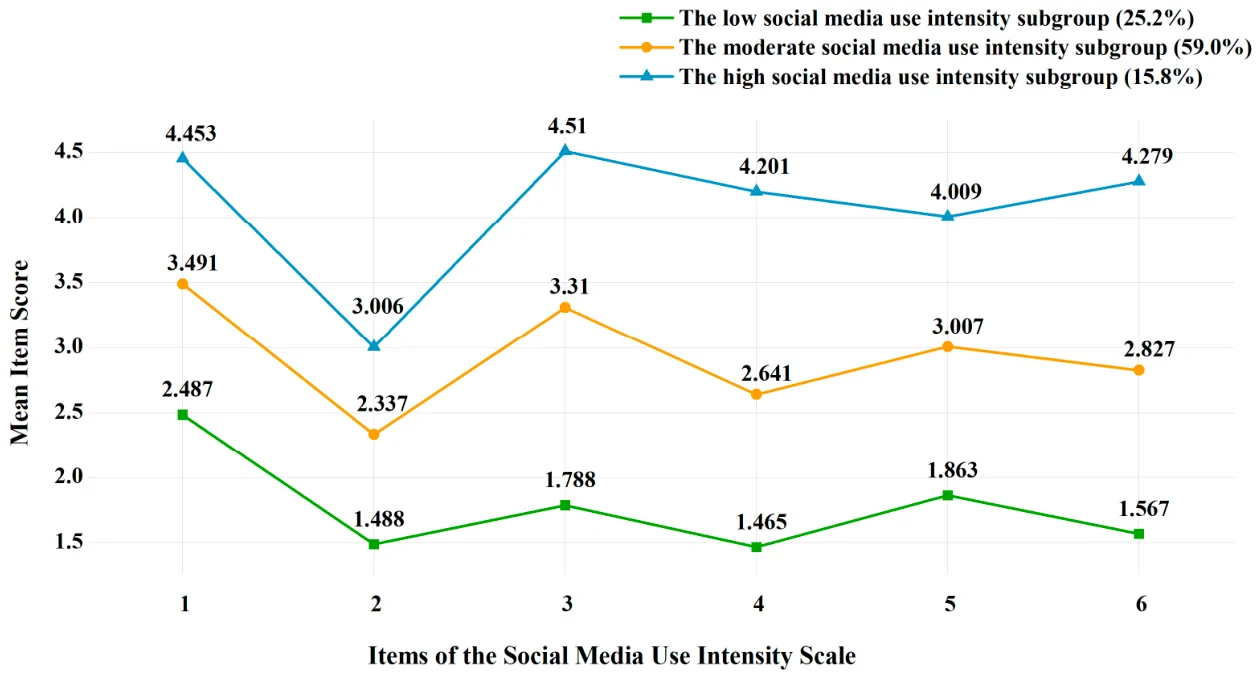
Mean Item Scores Across Adolescent Social Media Use Intensity Subgroups.

The mean scores of the three latent classes across the items of social media use intensity are displayed in Figure 2. The three classes showed similar patterns across the social media use intensity items, with differences primarily observed in the overall score levels. Specifically, Class 1 included 525 adolescents (25.2%). Adolescents in this profile showed relatively low scores across all social media use intensity items; therefore, this class was labeled the low social media use intensity subgroup. Class 2 included 1231 adolescents (59.0%). Adolescents in this profile demonstrated higher scores than Class 1, but lower scores than Class 3 across all social media use intensity items; accordingly, this class was labeled the moderate social media use intensity subgroup. Class 3 included 329 adolescents (15.8%). Adolescents in this profile exhibited relatively high scores across all social media use intensity items; therefore, this class was labeled the high social media use intensity subgroup.

### 3.3. Effects of Demographic Variables on Latent Classes of Adolescent Social Media Use Intensity

To further examine whether demographic variables were associated with latent profile membership, the R3STEP procedure in Mplus was used, with gender, age, and BMI included as covariates. The moderate social media use intensity subgroup was specified as the reference group. The associations between demographic variables and profile membership were evaluated based on odds ratios (ORs) and *p*-values, and the results are presented in Table 2. The results indicated that: (1) compared with the moderate social media use intensity subgroup, gender and age were significantly associated with membership in the low social media use intensity subgroup. Specifically, compared with males, females were less likely to belong to the low social media use intensity subgroup (OR = 0.608, *p* < 0.001), and older adolescents were also less likely to belong to this subgroup (OR = 0.698, *p* < 0.001), whereas BMI was not significantly associated with membership in the low versus moderate social media use intensity subgroups. (2) Compared with the moderate social media use intensity subgroup, age and BMI were significantly associated with membership in the high social media use intensity subgroup (OR = 1.171, *p* < 0.001; OR = 1.048, *p* < 0.05, respectively). Specifically, older adolescents and those with higher BMI were more likely to belong to the high social media use intensity subgroup.

Overall, gender, age, and BMI showed different patterns of associations with social media use intensity profiles. Higher age and BMI were associated with a greater likelihood of adolescents belonging to the high social media use intensity subgroup, suggesting that developmental characteristics and individual differences in body-related characteristics may be related to variations in adolescents’ social media use intensity. These findings suggest that older adolescents and those with higher BMI were more likely to belong to profiles characterized by stronger emotional connections with and greater integration of social media into daily life.

### 3.4. Differences in Psychological Indicators Across Adolescent Social Media Use Intensity Profiles

**Figure 3:**
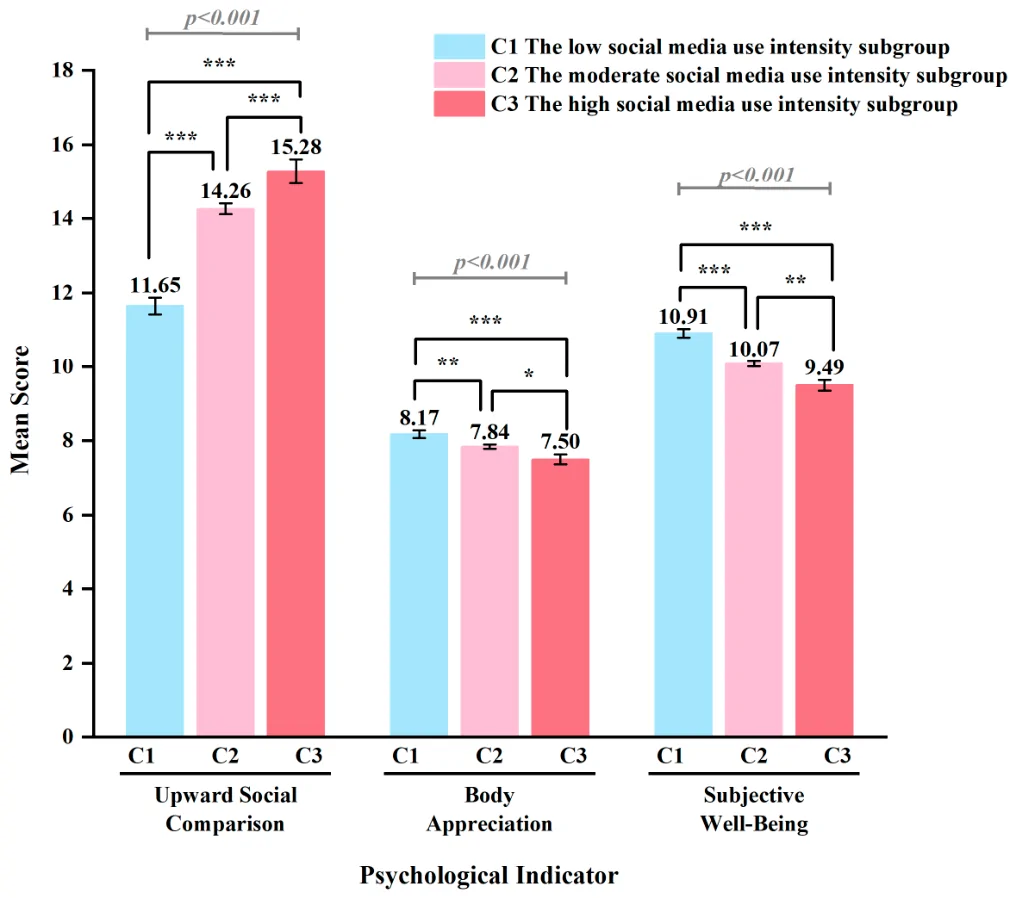
Psychological Differences Across Social Media Use Intensity Subgroups. Note: * *p* < 0.05, ** *p* < 0.01, *** *p* < 0.001. Error bars represent 95% confidence intervals.

Using the latent profile classifications obtained from LPA as the independent variable and adolescents’ scores on upward social comparison, body appreciation, and subjective well-being as outcome variables, further analyses were conducted to compare differences across social media use intensity profiles. The results are presented in Figure 3. The findings indicated that the three social media use intensity profiles differed significantly in scores on upward social comparison, body appreciation, and subjective well-being.

Specifically, (1) significant differences were observed among the three profiles in upward social comparison scores (Wald χ^2^ = 118.155, *p* < 0.001). The high social media use intensity subgroup showed the highest mean score for upward social comparison (M = 15.279), which was significantly higher than the scores of the low social media use intensity subgroup (M = 11.649) and the moderate social media use intensity subgroup (M = 14.256). Meanwhile, the moderate social media use intensity subgroup also showed significantly higher upward social comparison scores than the low social media use intensity subgroup, with the three subgroups showing a graded pattern of C3 > C2 > C1. (2) Significant differences were also observed among the three profiles in body appreciation scores (Wald χ^2^ = 17.306, *p* < 0.001). The low social media use intensity subgroup exhibited the highest level of body appreciation (M = 8.173), which was significantly higher than those of the moderate social media use intensity subgroup (M = 7.842) and the high social media use intensity subgroup (M = 7.504). The moderate social media use intensity subgroup also showed significantly higher body appreciation than the high social media use intensity subgroup, with the three subgroups showing a graded pattern of C1 > C2 > C3. (3) Significant differences were found among the three profiles in subjective well-being scores (Wald χ^2^ = 67.989, *p* < 0.001). The low social media use intensity subgroup reported the highest level of subjective well-being (M = 10.911), which was significantly higher than those of the moderate social media use intensity subgroup (M = 10.074) and the high social media use intensity subgroup (M = 9.493). The moderate social media use intensity subgroup also showed significantly higher subjective well-being than the high social media use intensity subgroup, with the three subgroups showing a graded pattern of C1 > C2 > C3.

### 3.5. Testing the Serial Mediation Model of Adolescent Social Media Use Intensity, Upward Social Comparison, Body Appreciation, and Subjective Well-Being

**Figure 4:**
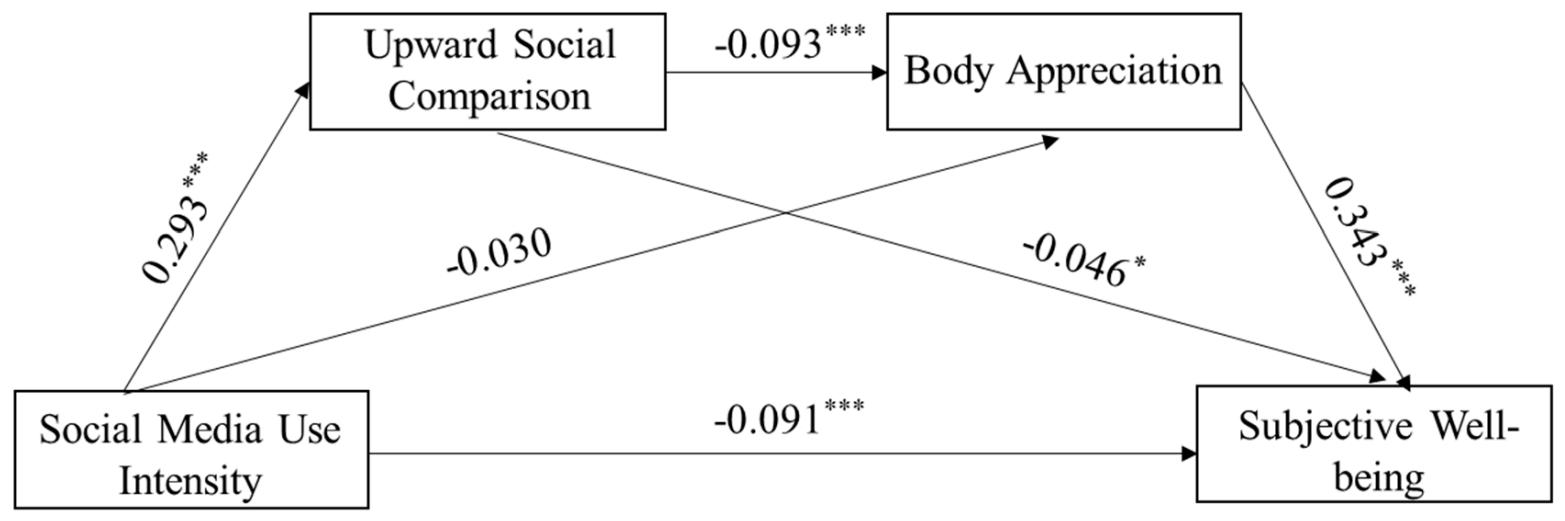
Sequential Mediation Model of the Association Between Social Media Use Intensity and Subjective Well-Being. Note: * *p* < 0.05, *** *p* < 0.001. Values represent standardized regression coefficients.

To investigate the potential mediating pathways between adolescent social media use intensity and subjective well-being, a serial mediation model was constructed with social media use intensity as the independent variable, subjective well-being as the dependent variable, and upward social comparison and body appreciation as mediators. Age, BMI, and gender were included as covariates, and the indirect effects were examined using a bootstrap approach. The results are presented in Figure 4, Table 3 and Table 4.

Regression analyses showed that social media use intensity was significantly positively associated with upward social comparison (β = 0.293, *p* < 0.001) and significantly negatively associated with subjective well-being (β = −0.091, *p* < 0.001). Upward social comparison was significantly negatively associated with body appreciation (β = −0.093, *p* < 0.001) and subjective well-being (β = −0.046, *p* < 0.05), whereas body appreciation was significantly positively associated with subjective well-being (β = 0.343, *p* < 0.001).

The mediation analysis indicated that the total effect (β = −0.124, 95% CI = [−0.170, −0.078]), direct effect (β = −0.091, 95% CI = [−0.136, −0.047]), and total indirect effect (β = −0.033, 95% CI = [−0.055, −0.011]) were all significant, as their confidence intervals did not include zero.

Specifically, the specific indirect effect through upward social comparison was significant (β = −0.013, 95% CI = [−0.027, −0.001]), and the specific sequential indirect effect through upward social comparison and body appreciation was also significant (β = −0.009, 95% CI = [−0.015, −0.005]).

Overall, the results indicated significant indirect effects through upward social comparison and the sequential pathway involving upward social comparison and body appreciation.

### 3.6. Multi-Group Analysis

**Figure 5:**
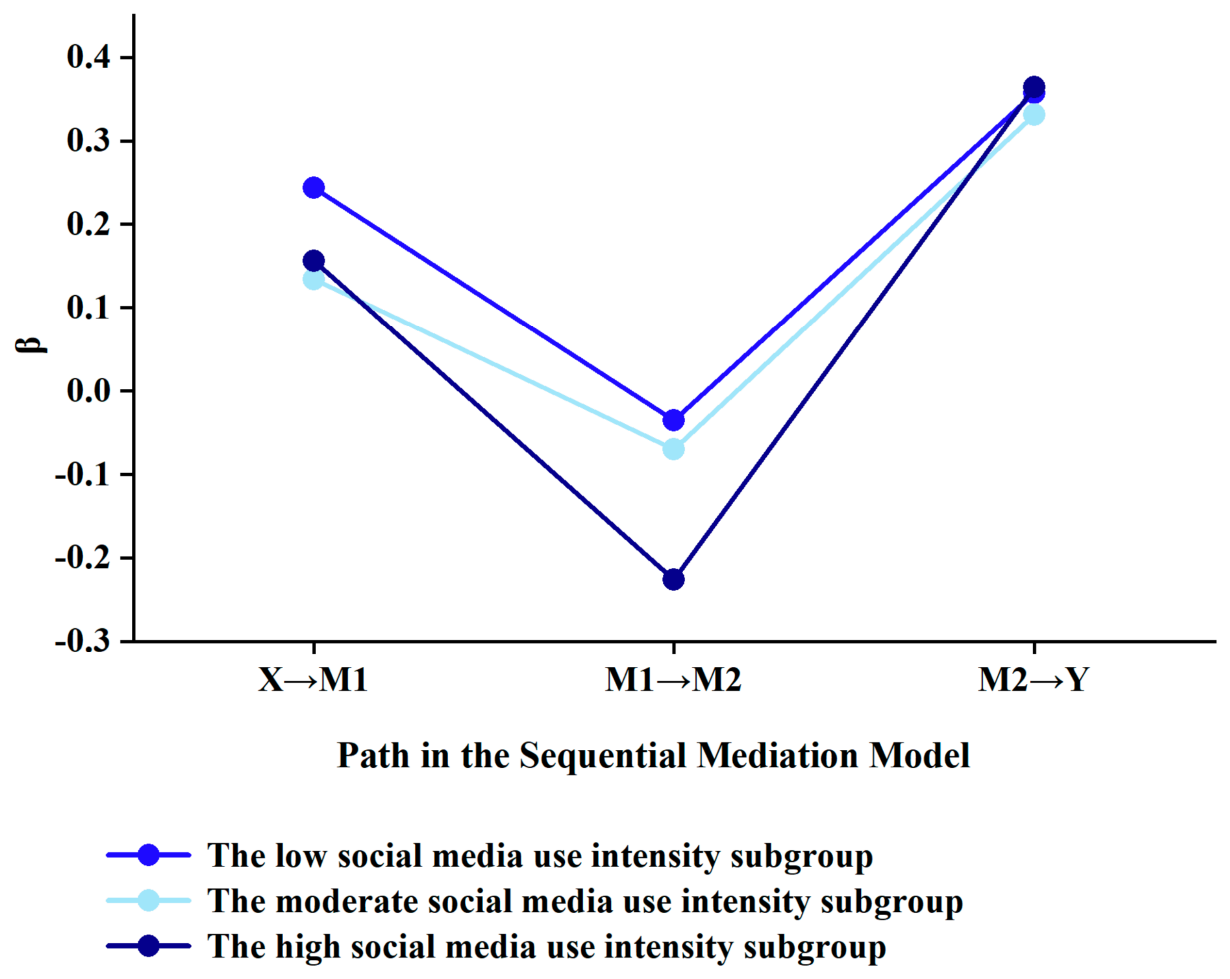
Standardized Path Coefficients Across Social Media Use Intensity Subgroups in the Sequential Mediation Model. Note: X = Social media use intensity; M1 = Upward social comparison; M2 = Body appreciation; Y = Subjective well-being.

**Figure 6:**
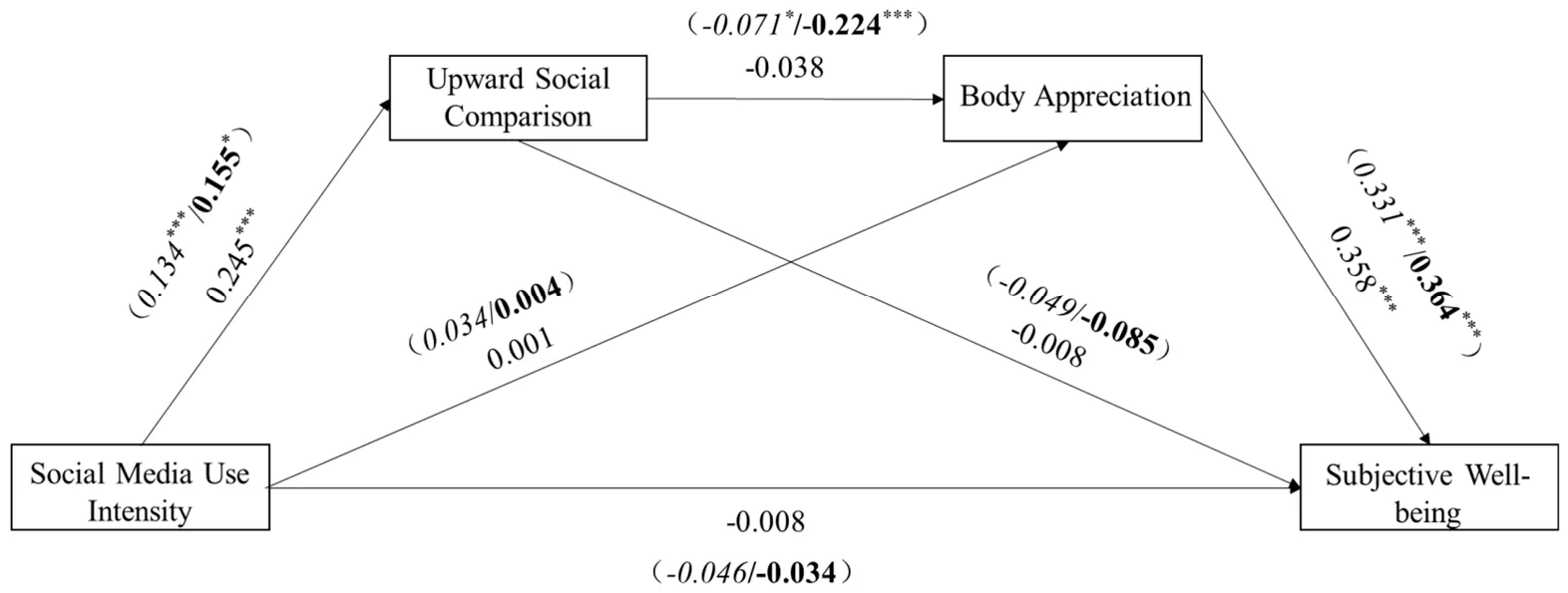
Sequential mediation model linking social media use intensity profiles to subjective well-being. Note: Values in regular font represent the Low Social media use intensity Profile, values in italicized font represent the Moderate Social media use intensity Profile, and values in bold font represent the High Social media use intensity Profile. * *p* < 0.05, *** *p* < 0.001.

To further examine whether the sequential mediation model was invariant across the low, moderate, and high social media use intensity subgroups, a multi-group analysis was conducted. Specifically, the chi-square difference test (Δχ^2^) was used to compare the unconstrained baseline model with the constrained model to examine the equality of path coefficients across groups. A significant chi-square difference test (*p* < 0.05) indicated that the corresponding path coefficients differed significantly between groups. The results are presented in Figure 5 and Figure 6.

Under the unconstrained model, subgroup-specific bootstrap analyses showed that the total effect, direct effect, and total indirect effect were all nonsignificant in both the low and moderate social media use intensity subgroups. In the high social media use intensity subgroup, these three effects were also nonsignificant; however, the specific sequential indirect effect through upward social comparison and body appreciation was significant (β = −0.013, 95% CI [−0.031, −0.003]). To further examine whether the structural paths differed across the three social media use intensity subgroups, chi-square difference tests were conducted. Under the fully constrained model, the results showed that the overall sequential mediation model did not differ significantly across the three subgroups (Δχ^2^ = 14.519, Δdf = 12, *p* = 0.269). However, the path coefficient for the association between upward social comparison and body appreciation differed significantly across the three subgroups (Δχ^2^ = 7.552, Δdf = 2, *p* = 0.023). Pairwise comparisons, with Bonferroni correction (adjusted α = 0.0167), showed that the high social media use intensity subgroup differed significantly from both the low social media use intensity subgroup (Δχ^2^ = 6.292, Δdf = 1, *p* = 0.012) and the moderate social media use intensity subgroup (Δχ^2^ = 6.179, Δdf = 1, *p* = 0.013) for this path. In contrast, the low social media use intensity subgroup and the moderate social media use intensity subgroup did not differ significantly in this path (Δχ^2^ = 0.291, Δdf = 1, *p* = 0.590). Based on the path coefficients, the negative association between upward social comparison and body appreciation was significantly stronger in the high social media use intensity subgroup (β = −0.224, *p* < 0.001) than in the low social media use intensity subgroup (β = −0.038, *p* > 0.05) and the moderate social media use intensity subgroup (β = −0.070, *p* < 0.05), whereas the low and moderate social media use intensity profiles did not differ significantly in this path (the specific statistical results are presented in the Appendix A).

Overall, the multi-group analysis demonstrated that the overall pattern of structural paths did not differ significantly across the three social media use intensity subgroups, with the exception of the path from upward social comparison to body appreciation, which differed significantly between the high social media use intensity subgroup and both the low and moderate social media use intensity subgroups.

## 4. Discussion

This study identified subgroups of adolescent social media use intensity using latent profile analysis in a sample of 2085 Chinese adolescents and further examined the associations among upward social comparison, body appreciation, and subjective well-being. The results indicated that gender, age, and BMI were significantly associated with adolescents’ membership in different social media use intensity profiles. Moreover, a significant sequential indirect pathway linking social media use intensity, upward social comparison, body appreciation, and subjective well-being was observed in the overall sample. However, the total effect, direct effect, and total indirect effect were not significant within any of the three profiles, whereas a significant specific sequential pathway was identified in the high social media use intensity subgroup. Furthermore, the negative association between upward social comparison and body appreciation was significantly stronger in the high social media use intensity subgroup than in both the low- and moderate-use intensity subgroups.

### 4.1. Profiles of Adolescent Social Media Use Intensity

Social media has become increasingly integrated into adolescents’ daily lives, serving not only as a context for social interaction but also as an important part of their everyday experiences and emotional lives. As adolescents may differ in the extent to which they are emotionally connected to and integrated with social media, examining individual differences in social media use intensity may provide a more nuanced understanding of adolescents’ social media experiences. Previous studies have primarily focused on social media use duration, frequency, and patterns, while variable-centered approaches have yielded inconsistent findings regarding the association between social media use and subjective well-being (Hancock et al., 2022; Jarman et al., 2021; Schemer et al., 2021). Therefore, the present study adopted a person-centered approach and conducted latent profile analysis to identify latent profiles of adolescents characterized by different patterns of social media use intensity. The results revealed three profiles of adolescent social media use intensity: the low social media use intensity subgroup, the moderate social media use intensity subgroup, and the high social media use intensity subgroup. These three profiles showed significant differences in social media use intensity scores, supporting Hypothesis 1.

Furthermore, gender, age, and BMI were significantly associated with adolescents’ membership in different social media use intensity profiles. Specifically, males were more likely than females to belong to the low social media use intensity subgroup, whereas older age and higher BMI were associated with a greater likelihood of belonging to the high social media use intensity subgroup. One possible interpretation is that the age range in the present sample (Grades 5–11) spans a period of substantial developmental transition, during which adolescents gradually shift their primary social contexts from family-oriented contexts toward increasingly peer-oriented social networks. Older adolescents may have greater access to personal digital devices and greater autonomy in their daily activities, which may provide more opportunities for social media engagement (Spies Shapiro & Margolin, 2014). In addition, the growing desire for independence and the increasing salience of peer relationships during this developmental period may also be associated with greater engagement with social media as a means of maintaining social connections. Similarly, adolescents with higher BMI may be more sensitive to appearance-related evaluations (Bucchianeri et al., 2013), which may be associated with greater attention to appearance-related information and social comparison processes on social media. However, these interpretations are not exhaustive, and the observed associations may also reflect other factors not assessed in the present study, such as physical activity, academic pressure, or parental mediation of technology use.

### 4.2. Differences in Psychological Indicators Among Adolescents with Different Social Media Use Intensity Profiles

The present study found that adolescents with different social media use intensity profiles differed significantly in upward social comparison, body appreciation, and subjective well-being. Compared with adolescents in the low and moderate social media use intensity subgroups, those in the high social media use intensity subgroup showed significantly higher levels of upward social comparison and significantly lower levels of body appreciation and subjective well-being, supporting Hypothesis 2. In addition, social media use intensity was positively associated with upward social comparison and negatively associated with body appreciation and subjective well-being, which is consistent with the propositions of social comparison theory (Buunk & Gibbons, 2007) and the tripartite influence model (Jarman et al., 2021; Jung et al., 2022) in social media research. As an important tool for adolescents to obtain information and engage in social interactions, social media provides extensive opportunities for adolescents to compare themselves with others. Compared with adolescents in the low and moderate social media use intensity subgroups, those in the high social media use intensity subgroup may have stronger emotional connections with social media and greater exposure to idealized appearance-related information, thereby encountering more opportunities for social comparison. These experiences may be associated with greater upward social comparison and lower levels of body appreciation and subjective well-being.

### 4.3. Interpretation of the Sequential Mediation Mechanism

In the overall sample, the significant sequential indirect pathway reflects a pattern of associations involving social media use intensity, upward social comparison, body appreciation, and subjective well-being. This pattern is broadly consistent with social comparison theory (Buunk & Gibbons, 2007) and the tripartite influence model (Jarman et al., 2021; Jung et al., 2022), which highlight the role of social comparison and appearance-related self-evaluation in adolescents’ body image. Adolescence represents a particularly important period for self-concept development and is also characterized by substantial physical development and changes in appearance, during which appearance-related standards may become increasingly salient. Social media provides adolescents with abundant social information and opportunities for self-evaluation, including information related to appearance and body ideals. Accordingly, greater social media use intensity may be associated with greater tendencies toward upward social comparison, which in turn may be associated with less favorable evaluations of one’s own body and lower body appreciation. Lower body appreciation has also been associated with poorer psychological well-being and more negative emotional experiences (Linardon et al., 2022). The sequential pattern is also broadly consistent with a cognitive–evaluative framework, in which upward social comparison represents a cognitive evaluative process, while body appreciation reflects a more affective evaluation of the self that may be linked to broader subjective well-being. Together, these associations may help explain the significant sequential pathway observed in the overall sample.

The multi-group findings provide further insight into this pattern. Although the overall sequential pathway was significant, the total effect, direct effect, and total indirect effect were not significant within any of the three social media use intensity subgroups. This discrepancy may partly reflect the reduced statistical power associated with subgroup analyses, but it may also indicate that the overall pattern does not manifest uniformly across social media use intensity profiles. Instead, the primary between-profile difference was observed in the association between upward social comparison and body appreciation, which was significantly stronger in the high social media use intensity subgroup than in the low and moderate social media use intensity subgroups. This pattern may reflect the greater psychological salience of social media among adolescents with high social media use intensity. Because social media use intensity in the present study reflects the extent to which adolescents are emotionally connected to social media and integrate it into their daily lives, higher intensity may indicate that social media experiences are more closely intertwined with adolescents’ self-evaluative processes. In such circumstances, appearance-related social comparisons may be more strongly associated with how adolescents evaluate their own bodies. At the same time, this association may be bidirectional. Adolescents with lower body appreciation or greater appearance concerns may be more likely to engage with social media in ways that are relevant to self-evaluation and social comparison. Previous longitudinal research has provided evidence for reciprocal associations between social media use, social comparison, and body dissatisfaction, suggesting that these processes may influence one another over time (Jarman et al., 2023; Rousseau et al., 2017). Thus, the stronger association between upward social comparison and body appreciation observed in the high social media use intensity subgroup may reflect a more closely intertwined relationship between social media experiences, social comparison, and body-related self-evaluation, rather than a simple unidirectional process.

The present findings suggest that families, schools, and society should pay attention to adolescents’ social media experiences, with particular attention to adolescents with high social media use intensity. Rather than simply encouraging adolescents to reduce social media use intensity, interventions could focus on helping them recognize and manage appearance-related social comparison in social media contexts. For example, positive body image education and media literacy programs could help adolescents critically evaluate appearance-related content and develop more balanced perceptions of their own bodies. Mindfulness-based approaches to social media use may also help adolescents become more aware of comparison-related thoughts and emotional responses. These approaches may provide practical avenues for supporting body appreciation and positive self-evaluations among adolescents.

### 4.4. Limitations and Future Research Directions

Several limitations should be acknowledged. First, the cross-sectional design prevents causal inferences regarding the relationships among the variables, and future studies should employ longitudinal or experimental designs to further clarify their temporal and causal relationships. Second, although gender, age, and BMI were included as covariates, the present study did not examine whether the associations and indirect pathways differed across gender. This is particularly relevant to research on body image, where gender differences may be important. In addition, the broad age range of 10–19 years encompasses substantial developmental differences in social media use, social comparison, body image, and subjective well-being, which may not be fully captured by treating age as a linear covariate. Future research should examine these associations across gender and developmental stages, and measurement invariance should be established before making comparisons across developmental groups. Third, all variables were assessed using self-report questionnaires, which may be influenced by common method bias and social desirability effects; future research could incorporate objective behavioral measures or ecological momentary assessment. Finally, although a significant sequential pathway was identified in the overall sample, its effect size was relatively small, and the subgroup analyses indicated that this pattern was not uniformly observed across the three profiles. Future research should incorporate additional individual and contextual factors, such as personality traits, social support, and self-esteem (Gerson et al., 2016; Hunt et al., 2018; Schemer et al., 2021; J.-L. Wang et al., 2017), and further examine targeted intervention approaches, such as media literacy programs, mindfulness-based approaches to social media use, and content-focused strategies, particularly for adolescents with high social media use intensity.

## 5. Conclusions

This study examined the associations between social media use intensity and adolescent subjective well-being by identifying latent profiles of social media use intensity and testing potential pathways involving upward social comparison and body appreciation. The findings indicated that (1) adolescents could be classified into three profiles based on their social media use intensity: the low social media use intensity profile, the moderate social media use intensity profile, and the high social media use intensity profile. Gender, age, and BMI were significantly associated with adolescents’ profile membership, with older adolescents and those with higher BMI being more likely to belong to the high social media use intensity profile. (2) Adolescents in the high social media use intensity profile showed the highest levels of social media use intensity and upward social comparison, while exhibiting the lowest levels of body appreciation and subjective well-being, indicating a pattern characterized by higher social media use intensity and upward social comparison, as well as lower body appreciation and subjective well-being. (3) Social media use intensity was negatively associated with adolescent subjective well-being, and a significant sequential pathway involving upward social comparison and body appreciation was observed in the overall sample, whereas the independent indirect effect of body appreciation was not significant.

Multi-group analysis showed no significant overall differences in structural paths across profiles, although the pathway from upward social comparison to body appreciation was significantly stronger in the high social media use intensity profile than in the low and moderate social media use intensity profiles. Overall, these findings highlight the associations among social media use intensity, upward social comparison, body appreciation, and subjective well-being, while suggesting that the association between upward social comparison and body appreciation may be particularly salient among adolescents with high social media use intensity.

## Figures and Tables

**Table 1:** Results of Latent Profile Analysis of Adolescent Social Media Use Intensity.

Number of Profiles	AIC	BIC	aBIC	Entropy	LMR-LRT	BLRT	Class Proportion
2	35,822.891	35,930.099	35,869.735	0.775	0.000	0.000	39.4%/60.6%
3	34,717.439	34,864.145	34,781.540	0.815	0.000	0.000	25.2%/59.0%/15.8%
4	34,396.811	34,583.015	34,478.171	0.775	0.015	0.000	9.1%/49.7%/28.9%/12.3%
5	34,119.978	34,345.679	34,218.595	0.737	0.000	0.000	26.8%/9.0%/25.5%/25.0%/13.9%

**Table 2:** Demographic Characteristics Associated with Social Media Use Intensity Profile Membership.

Predictor	The Low Social Media Use Intensity Subgroup		The High Social Media Use Intensity Subgroup	
	β (95% CI)	OR (95% CI)	β (95% CI)	OR (95% CI)
Gender	−0.497 *** [−0.742,−0.252]	0.608 *** [0.476,0.777]	0.084 [−0.201,0.370]	1.088 [0.818,1.448]
Age	−0.359 *** [−0.441,−0.278]	0.698 *** [0.643,0.758]	0.158 *** [0.075,0.241]	1.171 *** [1.078,1.273]
BMI	−0.021 [−0.064, 0.022]	0.979 [0.938,1.022]	0.046 * [0.004,0.089]	1.048 * [1.004,1.093]

Note: The reference group was the moderate social media use intensity subgroup. * *p* < 0.05, *** *p* < 0.001.

**Table 3:** Regression analysis of the sequential mediation model.

Predictor	Model 1 (Upward Social Comparison)		Model 2 (Body Appreciation)		Model 3 (Subjective Well-Being)	
	β	t	β	t	β	t
Social media use intensity	0.293	12.502 ***	−0.030	−1.171	−0.091	−3.935 ***
Upward social comparison			−0.093	−3.820 ***	−0.046	−2.057 *
Body appreciation					0.343	15.861 ***
R^2^	0.080		0.028		0.202	
F	6.312 ***		3.411 **		11.616 ***	

Note: Standardized regression coefficients (β) are reported. * *p* < 0.05, ** *p* < 0.01, *** *p* < 0.001.

**Table 4:** Bootstrap test of the sequential mediation effect.

Effect Type	Effect	Boot SE	Boot 95% CI	
			Lower Limit	Upper Limit
Total effect	−0.124	0.024	−0.170	−0.078
Direct effect	−0.091	0.023	−0.136	−0.047
Total indirect effect	−0.033	0.011	−0.055	−0.011
1-2-4	−0.013	0.007	−0.027	−0.001
1-3-4	−0.010	0.009	−0.028	0.007
1-2-3-4	−0.009	0.003	−0.015	−0.005

Note: 1 = social media use intensity, 2 = upward social comparison, 3 = body appreciation, and 4 = subjective well-being.

## Data Availability

The data that support the findings of this study are available from the corresponding author upon reasonable request. The data are not publicly available due to privacy and ethical restrictions.

## Appendix A

**Table A1:** Regression Analysis of the Sequential Mediation Model in the Low Social Media Use Intensity Subgroup.

Predictor	Model 1 (Upward Social Comparison)		Model 2 (Body Appreciation)		Model 3 (Subjective Well-Being)	
	β	t	β	t	β	t
Social Media Use Intensity	0.245	5.954 ***	0.001	0.012	−0.008	−0.186
Upward Social Comparison			−0.038	−0.878	−0.008	−0.199
Body Appreciation					0.358	8.580 ***
R^2^	0.072		0.010		0.185	
F	3.339 **		0.873		5.348 ***	

Note: Gender, age, and BMI were included as covariates, β represents the standardized regression coefficient. ** *p* < 0.01, *** *p* < 0.001.

**Table A2:** Bootstrap Analysis of the Sequential Mediation Effects in the Low Social Media Use Intensity Subgroup.

Effect Type	Effect	Boot SE	Boot 95% CI	
			Lower Limit	Upper Limit
Total Effect	−0.013	0.044	−0.095	0.075
Direct Effect	−0.008	0.043	−0.090	0.078
Total Indirect Effect	−0.005	0.020	−0.045	0.032
1-2-4	−0.002	0.010	−0.023	0.018
1-3-4	0.000	0.017	−0.036	0.031
1-2-3-4	−0.003	0.004	−0.012	0.004

Note: 1 = social media use intensity, 2 = upward social comparison, 3 = body appreciation, 4 = subjective well-being.

**Table A3:** Regression Analysis of the Sequential Mediation Model in the Moderate Social Media Use Intensity Subgroup.

Predictor	Model 1 (Upward Social Comparison)		Model 2 (Body Appreciation)		Model 3 (Subjective Well-Being)	
	β	t	β	t	β	t
Social Media Use Intensity	0.134	4.261 ***	0.034	1.084	−0.046	−1.496
Upward Social Comparison			−0.071	−2.329 *	−0.049	−1.730
Body Appreciation					0.331	11.366 ***
R^2^	0.021		0.030		0.161	
F	2.141 *		2.931 **		7.517 ***	

Note: Gender, age, and BMI were included as covariates, β represents the standardized regression coefficient. * *p* < 0.05, ** *p* < 0.01, *** *p* < 0.001.

**Table A4:** Bootstrap Analysis of the Sequential Mediation Effects in the Moderate Social Media Use Intensity Subgroup.

Effect Type	Effect	Boot SE	Boot 95% CI	
			Lower Limit	Upper Limit
Total Effect	−0.045	0.033	−0.103	0.028
Direct Effect	−0.046	0.031	−0.101	0.021
Total Indirect Effect	0.001	0.012	−0.023	0.023
1-2-4	−0.007	0.005	−0.016	0.002
1-3-4	0.011	0.010	−0.011	0.030
1-2-3-4	−0.003	0.002	−0.007	0.000

Note: 1 = social media use intensity, 2 = upward social comparison, 3 = body appreciation, 4 = subjective well-being.

**Table A5:** Regression Analysis of the Sequential Mediation Model in the High Social Media Use Intensity Subgroup.

Predictor	Model 1 (Upward Social Comparison)		Model 2 (Body Appreciation)		Model 3 (Subjective Well-Being)	
	β	t	β	t	β	t
Social Media Use Intensity	0.155	2.482 *	0.004	0.057	−0.034	−0.557
Upward Social Comparison			−0.224	−3.821 ***	−0.085	−1.460
Body Appreciation					0.364	6.681 ***
R^2^	0.031		0.087		0.219	
F	1.368		2.707 **		4.978 ***	

Note: Gender, age, and BMI were included as covariates, β represents the standardized regression coefficient. * *p* < 0.05, ** *p* < 0.01, *** *p* < 0.001.

**Table A6:** Bootstrap Analysis of the Sequential Mediation Effects in the High Social Media Use Intensity Subgroup.

Effect Type	Effect	Boot SE	Boot 95% CI	
			Lower Limit	Upper Limit
Total Effect	−0.058	0.066	−0.189	0.070
Direct Effect	−0.034	0.060	−0.151	0.088
Total Indirect Effect	−0.024	0.028	−0.083	0.027
1-2-4	−0.013	0.011	−0.043	0.003
1-3-4	0.001	0.023	−0.044	0.045
1-2-3-4	−0.013	0.007	−0.031	−0.003

Note: 1 = social media use intensity, 2 = upward social comparison, 3 = body appreciation, 4 = subjective well-being.

**Table A7:** Results of Multigroup Analysis.

Constrained Path	χ^2^	df	Δχ^2^	Δdf	*p*
Unconstrained baseline model	0	0			
Fully constrained model	14.519	12	14.519	12	0.269
Constraint on a1 equality	3.656	2	3.656	2	0.161
Constraint on a3 equality	7.552	2	7.552	2	0.023
Constraint on b2 equality	0.003	2	0.003	2	0.998

Note: a1: Social media use intensity → Upward social comparison; a3: Upward social comparison → Body appreciation; b2: Body appreciation → Subjective well-being.

**Table A8:** Multi-group Comparison of the Path from Upward Social Comparison to Body Appreciation.

Path Comparison	χ^2^	df	Δχ^2^	Δdf	*p*
Low-use intensity subgroup vs. Moderate-use intensity subgroup	0.291	1	0.291	1	0.590
Low-use intensity subgroup vs. High-use intensity subgroup	6.292	1	6.292	1	0.012
Moderate-use intensity subgroup vs. High-use intensity subgroup	6.179	1	6.179	1	0.013

Note: The Bonferroni correction was applied to control the Type I error rate associated with multiple comparisons, with the significance level adjusted to 0.0167 (0.05/3).
